# Identification of Critical Variables and Critical Gap Variables in Hospital Nurses’ Job Satisfaction During the Dynamic Adjustment Phase of COVID‐19 Prevention in China: A Hybrid Machine Learning and Decision Analysis Approach

**DOI:** 10.1155/jonm/7240298

**Published:** 2026-04-29

**Authors:** Fuqin Tang, Lili Feng, Siqi Liu, Mao Ye, Chao Liu, Yen-Ching Chuang, Weifang Xu

**Affiliations:** ^1^ Nursing Department, Taizhou Central Hospital (Taizhou University Hospital), Taizhou, China, tzc.edu.cn; ^2^ Department of Intensive Care Unit, Taizhou Central Hospital (Taizhou University Hospital), Taizhou, 318000, Zhejiang, China, tzc.edu.cn; ^3^ Medical Department, The Second Affiliated Hospital of Hebei North University, Zhangjiakou, 075000, Hebei, China; ^4^ Hebei Research Center for Population Health Informatization and Technological Advancement, Zhangjiakou, 075000, Hebei, China; ^5^ Institute of Public Health and Emergency Management, Taizhou University, Taizhou, Zhejiang, China, tzc.edu.cn; ^6^ Business College, Taizhou University, Taizhou, Zhejiang, China, tzc.edu.cn; ^7^ Department of Orthopedics, Taizhou Central Hospital (Taizhou University Hospital), Taizhou, 318000, Zhejiang, China, tzc.edu.cn

**Keywords:** dynamic adjustment phase of COVID-19 prevention, hospital nurse, importance-performance analysis, nurses’ job satisfaction, random forest

## Abstract

**Purpose:**

This study aims to analyze the critical variables and gap variables affecting hospital nurses’ job satisfaction and propose improvement strategies based on the knowledge domains of nursing decision‐makers.

**Methods:**

This study, conducted between September and October 2022 during the dynamic adjustment phase of COVID‐19 prevention and control in China, was based on the McCloskey/Mueller Satisfaction Scale (MMSS) and developed a hybrid machine learning and decision analysis tool model. The random forest (RF) method was used to estimate the importance of each variable in the data, and the importance‐performance analysis (IPA) was used to identify critical gap variables and propose improvement strategies.

**Results:**

The RF analysis (OOB error rate = 17.93%) identified “Decision‐making” (*C*_30_, importance score = 0.053) and “Control‐work conditions” (*C*_29_, importance score = 0.067) as the most influential factors (critical variables) in determining nurses’ job satisfaction. The IPA analysis identified *C*_30_ as the most critical gap variable, indicating a significant need to improve nurses’ involvement in hospital decision‐making processes.

**Conclusions:**

To improve nurse job satisfaction and retention, hospital decision‐makers and nursing departments should implement policies that enhance nurses’ involvement in decision‐making, particularly those with experience in pandemic‐related healthcare challenges. Addressing these factors could foster a more supportive and resilient nursing work environment.

## 1. Introduction

Nurses’ job satisfaction is a key determinant of workforce stability, quality of care, and patient safety. Low job satisfaction has been associated with increased burnout, turnover intention, and reduced care quality, whereas higher satisfaction contributes to better retention and improved healthcare outcomes [1–3]. Despite sustained attention, nurse retention remains a global challenge, and improving job satisfaction continues to be a priority in healthcare management.

Even before the novel coronavirus (COVID‐19) pandemic, shortages in the nursing workforce were widely reported. The pandemic has further exacerbated this issue, leading to increased attrition rates and intensified workforce pressures [4]. The International Council of Nurses reported that in January 2021, more than 130 National Nurses’ Associations anticipated a potential mass exodus from the profession [5]. Similarly, data from the Current Population Survey indicated that within a single year, approximately 100,000 nurses left the workforce, including a 4% decline among those under the age of 35 [6]. These trends highlight the urgent need to better understand and improve nurses’ job satisfaction.

Previous studies have examined factors related to nursing outcomes, primarily focusing on work stress [7, 8], mental health issues [9], job burnout [10], and intention to leave the profession [11]. In addition, nurse shortages have been shown to negatively affect the quality of patient care [2, 12]. However, the relative importance of different job satisfaction factors and their prioritization in complex healthcare settings remains insufficiently explored.

The COVID‐19 pandemic has introduced additional challenges to nursing practice [13]. As frontline healthcare professionals, nurses have played a central role in both patient care and infection control [14, 15]. As the world transitions to a “new normal” in COVID‐19 prevention and control, ensuring the protection and well‐being of healthcare workers remains a priority [16]. In China, the “new normal” of COVID‐19 prevention refers to the dynamic adjustment phase that began in mid‐to‐late 2022. During this period, hospitals transitioned from full emergency response to a hybrid model integrating routine care with flexible outbreak management strategies. Nurses are required to perform routine clinical duties while simultaneously undertaking infection prevention tasks, such as nucleic acid testing, temperature monitoring, and administrative coordination [17]. These expanded responsibilities have increased both physical workload and psychological stress [18].

This transitional phase introduces unique operational characteristics that differ from earlier stages of the pandemic. However, limited research has examined how nurses’ job satisfaction is shaped under these evolving conditions. Therefore, it is necessary to identify key determinants of job satisfaction and prioritize areas for improvement within this specific context.

## 2. Literature Review

One of the most widely used and validated instruments for measuring nurses’ job satisfaction is the McCloskey/Mueller Satisfaction Scale (MMSS). Scholars have employed the MMSS to evaluate nurse job satisfaction using the three primary methodological approaches:

I. Statistical analysis methods: These studies predominantly utilize descriptive statistics or inferential statistical techniques to examine relationships between variables. For example, Wang et al. [19] investigated the correlation between nursing handover quality, job satisfaction, and group cohesion among psychiatric nurses. Said and El‐Shafei [20] used the MMSS to analyze job satisfaction, occupational stress, and turnover intention in 210 nurses from one of Egypt’s COVID‐19 triage hospitals. Du et al. [21] conducted a large‐scale study involving 6158 nurses across 34 hospitals in China, evaluating factors influencing job satisfaction. In a related study, Lea et al. [22] employed structural equation modeling to investigate the levels of burnout, job satisfaction, and turnover intention among Certified Registered Nurse Anesthetists (CRNAs) during the COVID‐19 surges. Although widely adopted, statistical methods mainly focus on correlation rather than prediction. They often lack the capacity to capture nonlinear patterns or to rank variable importance in complex work environments.
II. Decision analysis methods: These methods integrate expert judgment and operations research techniques to develop quantitative models for assessing nurse satisfaction. Wang et al. [23] proposed a consistent fuzzy preference relation (CFPR) analysis and an importance‐performance analysis (IPA) model to evaluate and improve home healthcare nurses’ job satisfaction. Liu et al. [24] applied the decision‐making trial and evaluation laboratory (DEMATEL) and IPA methods to evaluate and enhance nurse satisfaction. Compared to statistical approaches, decision analysis models are less frequently used in studies of nursing job satisfaction, which represents a potential research gap in the field [23–25].
III. Emerging analytical approaches: In recent years, machine learning and mixed‐method approaches have gained attention as promising alternatives to traditional methods. Specifically, techniques such as random forest (RF) provide nonparametric models with high interpretability, allowing for robust ranking of variable importance and improved generalizability. For instance, Chen et al. [26] employed neural networks to analyze turnover intention among healthcare staff. Jura et al. [27] used sentiment analysis and clustering analysis to assess the job satisfaction of registered nurses. By integrating these emerging analytical tools, researchers can overcome the limitations of traditional statistics and expert‐based tools, as these approaches offer data‐driven, scalable solutions.

This study addresses the above methodological gaps by proposing a hybrid analytical framework that combines (1) the feature importance quantification capability of RF and (2) the managerial interpretability of IPA analysis. This integration enables the systematic identification of both critical satisfaction and critical gap variables in a real‐world hospital setting.

Importantly, few existing studies have contextualized findings across the full COVID‐19 pandemic timeline. During the initial outbreak stage, research focused largely on acute stress, emergency workload, and fear of infection. As the pandemic progressed, studies shifted to emphasize long‐term workload, personal protective equipment (PPE) access, and burnout prevention strategies. In contrast, the dynamic adjustment phase, marked by the easing of emergency protocols yet continued policy shifts and operational strain, has been relatively overlooked.

During this transition, nurses face dual responsibilities of routine care and infection control under evolving institutional policies. Their work characteristics, stressors, and determinants of satisfaction are likely to differ from those in earlier stages. However, few studies have adopted structured prioritization models to analyze these dynamics in this phase. Our study specifically addresses this neglected research gap by applying a hybrid model to evaluate nurses’ job satisfaction during the dynamic adjustment phase of COVID‐19 prevention and control in China. Table 1 summarizes key studies, their methodologies, findings, and limitations.

**TABLE 1:** Comparison of different analytical models of previous studies.

Category	Authors	Research methods	Key findings	Limitations
Statistical methods	Wang et al. [19]	Descriptive statistics, Pearson’s correlation, and hierarchical regression analysis	Job satisfaction mediates the relationship between group cohesion and handover quality	Cross‐sectional design limits causal inferences; self‐report bias; limited sample size
	Said and El‐Shafei [20]	Descriptive statistics, chi‐square test, and binary logistic regression	Nurses in COVID‐19 triage hospitals experience higher occupational stress and lower job satisfaction compared to those in general hospitals. Workload, biosecurity measures, and stigma are major stressors	Excluded nurses from isolation hospitals; single‐item measure for intent to leave; limited hospital types, affecting generalizability
	Du et al. [21]	Descriptive statistics, bivariate logistic analysis, and nonparametric tests	Missed nursing care (MNC) frequently occurs due to sudden increases in workload. Human resource issues are a major reason for MNC	Convenience sampling limits generalizability; survey distribution biases
	Lea et al. [22]	Structural equation modeling	A decrease in feedback, low CRNA‐administration relations scores, and prioritizing work over personal responsibilities were predictive of CRNA burnout. In addition, burnout levels were correlated with job satisfaction and turnover intention	A relatively small sample size and limited validity testing of the CRNA Organizational Climate Questionnaire were some limitations of this study

Decision‐making methods	Wang et al. [23]	Consistent fuzzy preference relation and importance‐performance analysis	Key factors influencing home healthcare nurses′ job satisfaction include salary, benefits, and peer relationships	Based on the CFPR method, assuming independent attributes, a small sample size (31 nurses) from one region
	Liu et al. [24]	Decision‐making trial and evaluation laboratory and importance‐performance analysis	Salary and benefits have the highest weight in job satisfaction. Leadership, support, and work conditions significantly impact nurse retention	Small sample size (15 nursing specialists); lack of comparison with other MCDM methods

Emerging analytical approaches	Chen et al. [26]	Artificial neural networks	A 24‐item ANN model with 53 parameters estimated by the ANN was developed to improve the accuracy of nurses’ intention to quit their jobs	There is no evidence to support that it is suitable for novice nurses in other countries/regions
	Jura et al. [27]	Sentiment analysis and clustering analysis	Sentiment analysis scores were significantly associated with the job satisfaction groups in both bivariate and multivariate analyses	The association analysis between sentiment scores and satisfaction clusters is limited to a subsample of the study cohort

## 3. Materials and Methods

### 3.1. Research Design and Modeling Process

**FIGURE 1:**
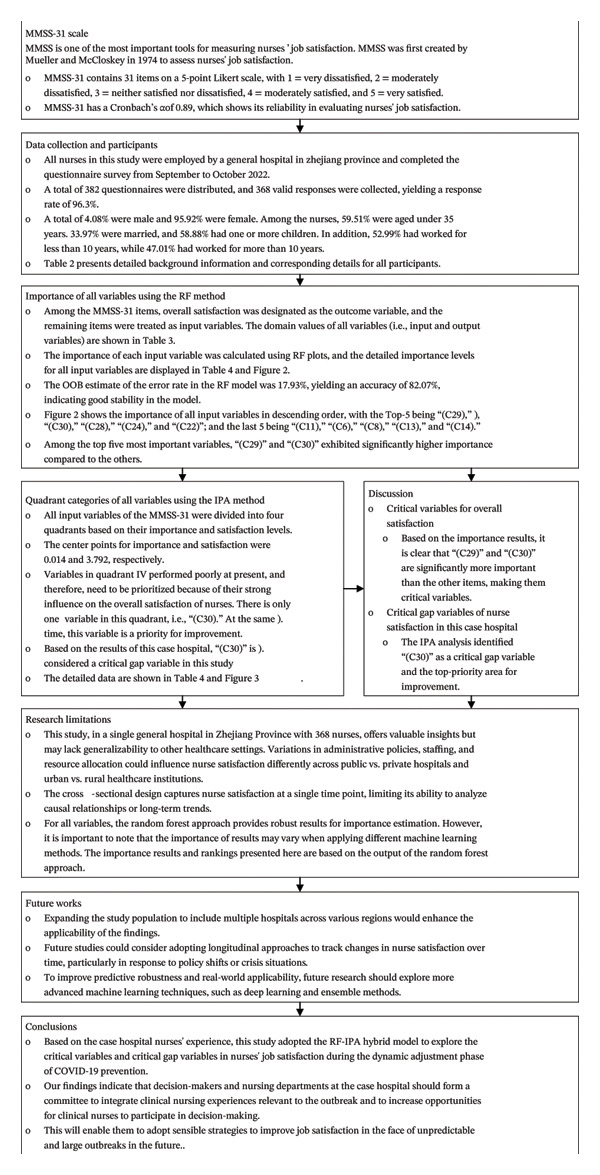
Research design and modeling process.

First, 368 nurses in the case hospital were questioned using the MMSS‐31. Second, RF analysis was used to estimate the importance of each variable and identify the critical variables. Finally, the IPA method was used to analyze the feature category of each satisfaction item and identify the critical gap variables with the highest priority for improvement. The research design and modeling process of this study is presented in Figure 1.

### 3.2. Ethics Approval and Participant Confidentiality

This study was reviewed and approved by the Ethics Committee of Taizhou Central Hospital (Taizhou University Hospital) under approval number: 2022L‐09‐28. All procedures involving human participants were conducted in accordance with the ethical standards of the institutional research committee and the principles outlined in the Declaration of Helsinki. Written informed consent was waived by the ethics committee due to the survey’s noninterventional, anonymous nature. Prior to participation, all respondents were fully informed about the purpose and content of the study, and voluntary completion of the questionnaire was considered to constitute oral informed consent.

### 3.3. MMSS‐31

MMSS is one of the most important tools for measuring nurses’ job satisfaction. MMSS was first created by Mueller and McCloskey in 1974 to assess nurses’ job satisfaction [28]. The MMSS‐31 was created based on the original MMSS evaluation system [29]. MMSS‐31 contains 31 items on a 5‐point Likert scale, with 1 = very dissatisfied, 2 = moderately dissatisfied, 3 = neither satisfied nor dissatisfied, 4 = moderately satisfied, and 5 = very satisfied. MMSS‐31 has a Cronbach’s *α* of 0.89, which shows its reliability in evaluating nurses’ job satisfaction. MMSS‐31 is used to measure job satisfaction among different categories of nurses, including nurses working on the front lines of the COVID‐19 pandemic [20], psychiatric nurses [19], nurse‐midwives [30], registered nurses [31], and Chinese nurses [32].

### 3.4. RF Method

The RF method, a robust algorithm that integrates the prediction results of weak learners (i.e., decision trees), is based on bootstrap sample technology [33]. The algorithm uses the bootstrap repeated sampling technique; therefore, it can generate decision trees under different conditions, which helps reduce the risk of overfitting [34]. Robust predictions and project importance are the main features of this method; therefore, it is widely used across various fields, including agriculture [35], industry [36], and medical genetics [37]. The procedure of the RF method is detailed in Breiman [33]. The general implementation steps of the method are as follows:

 Step 1: The training samples for each decision tree are generated using the bootstrap sampling method, which creates different training sets from the original dataset by randomly sampling with replacement. In addition, bootstrap sampling leaves approximately one‐third of the original dataset out of the training set, resulting in about 36.8% of the dataset being used as a test sample [38]. This method ensures that each decision tree is trained on a slightly different subset of data, thus increasing the diversity and generalizability of the model.
 Step 2: Predictive models are trained using the test samples, which are dedicated to evaluating the performance of the model and calculating its prediction error, referred to as out‐of‐bag (OOB) errors. Notably, cross‐validation was not used as a secondary accuracy verification method in this study. The rationale for this decision is discussed in the following sections [38, 39]:
 First, the OOB method is conceptually similar to traditional cross‐validation techniques. Both methods involve dividing the original dataset into multiple subdatasets. These subdatasets are alternately used as training sets, while the remaining data (i.e., data not used for training) are employed as test sets. However, a key difference between the two approaches lies in the way the data are divided: cross‐validation splits the dataset into several equal‐sized parts, ensuring that no sample appears more than once in the training set. In contrast, the bootstrap sampling process used in the OOB method may select the same sample multiple times for inclusion in the training set. Despite this difference, the error rate from the OOB method, using the OOB sample as the test set, is comparable to that from cross‐validation. This makes the OOB error a useful estimate of the model’s generalization error.
 Step 3: In this step, the importance of a variable is estimated by randomly permuting its assignments and observing the change in the model’s predictive accuracy. The “Mean decrease accuracy” metric quantifies the importance of each variable, with larger values indicating greater importance. A significant decrease in accuracy after permuting a variable indicates that the variable plays a critical role in the model’s predictive power.

The steps outlined above were implemented in this study using the “RandomForest” package (Version 4.7‐1.1) in the R programming language.

### 3.5. IPA Method

The IPA method was first described in the marketing literature [40]; however, it applies well to nursing. IPA divides all items into categories and corresponding decision‐making views, enabling nursing decision‐makers to rapidly understand the current satisfaction situation and propose appropriate improvement strategies [24]. Therefore, studies in health and nursing, such as health perception [41], patient care [42], and intensive care research coordination [43], have also employed this method. In this study, importance was plotted on the *x*‐axis (representing the importance of the RF output), while performance (satisfaction) was plotted on the *y*‐axis, dividing the data into four quadrants. The four quadrants and their corresponding strategic perspectives are described as follows [23, 24, 44]:

 Keep up the good work (Quadrant I): Items in this quadrant are rated high in both importance and performance. They represent current strengths in the work environment, such as peer support or effective communication, and should be sustained through continued investment. *Recommendation: Decision-makers should maintain the performance levels of these initiatives, as they represent satisfaction-related factors valued by nurses at the case hospital and currently contribute to relatively high levels of nurse satisfaction.*
 Possible overkill (Quadrant II): These items exhibit high performance but low importance to nurses. While they currently function well, they may not significantly influence overall satisfaction. *Recommendation: Decision-makers may consider reallocating resources from these items if constraints are in place.*
 Low priority (Quadrant III): These items are underperforming and of low importance to nurses. While they currently function poorly, they may not significantly influence overall satisfaction. *Recommendation: Given limited resources, decision-makers should postpone addressing these projects or lower their priority in near-term improvement efforts.*
 Concentrated here (Quadrant IV): This is the most critical quadrant, containing variables that are highly important yet underperforming. These items, referred to in this study as critical gap variables, are pivotal to satisfaction but currently fail to meet expectations. Improving performance in these areas is essential for maximizing impact on overall satisfaction. *Recommendation: Given limited resources, decision-makers should prioritize improving the performance of these items. They are currently the focus of nurses’ attention and represent the most immediate opportunities for enhancing satisfaction.*

The above four categories (i.e., Quadrants I–IV) provide more than theoretical insight; they serve as actionable guidance for nursing administrators seeking to optimize job satisfaction under resource constraints.

## 4. Results

### 4.1. Data Collection and Participants

All nurses in this study were employed by a general hospital in Zhejiang Province and completed the questionnaire survey from September to October 2022. During this time, the hospital was normalizing the prevention and control of the COVID‐19 pandemic; that is, in addition to general medical treatment and nursing measures, staff also conducted nucleic acid testing, provided emergency treatment, and implemented support measures for COVID‐19.

A total of 382 questionnaires were distributed, and 368 valid responses were collected, yielding a response rate of 96.3%. A total of 4.08% were male and 95.92% were female. Among the nurses, 59.51% were aged under 35 years, 33.97% were married, and 58.88% had one or more children. In addition, 52.99% had worked for less than 10 years, while 47.01% had worked for more than 10 years. Table 2 presents detailed background information and corresponding details for all participants.

**TABLE 2:** The background description of 368 nurses in the case hospital.

Characteristics	Number	Value (%)
Sex		
Male	15	4.08
Female	353	95.92
Age		
< 35	219	59.51
35–44	133	36.14
≥ 45	16	4.35
Education		
Junior college	49	13.31
Bachelor	314	85.33
Master and above	5	1.36
Professional title		
Junior nurse	167	45.38
Supervisor nurse	141	38.32
Senior nurse	18	4.89
Other	42	11.41
Marriage		
Married	125	33.97
Unmarried	241	65.49
Other	2	0.54
Number of children		
None	155	42.12
One	121	33.88
Multiple	92	25.00
Years of service		
Under 10 years	195	52.99
11–19 years	113	30.71
20 and above	60	16.30

### 4.2. Importance of all Variables Using the RF Method

**FIGURE 2:**
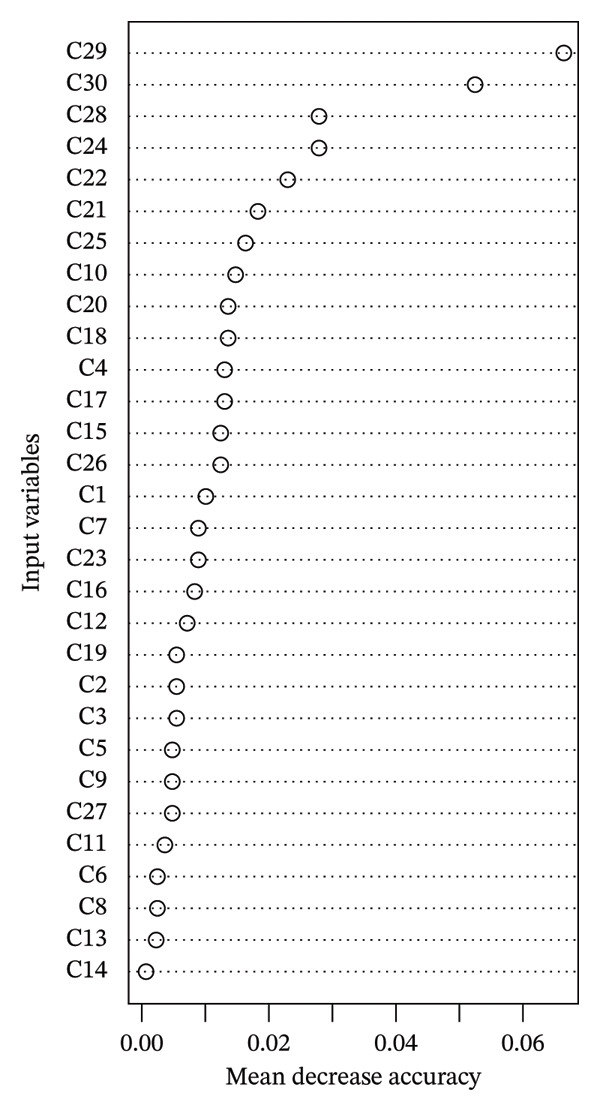
The importance of each input variable.

Among the MMSS‐31 items, overall satisfaction was designated as the outcome variable, and the remaining items were treated as input variables. The domain values of all variables (i.e., input and output variables) are shown in Table 3. The importance of each input variable was calculated using RF plots, and the detailed importance levels for all input variables are displayed in Table 4 and Figure 2. The OOB estimate of the error rate in the RF model was 17.93%, yielding an accuracy of 82.07%, indicating good stability in the model.

**TABLE 3:** The MMSS‐31 Scale with domain values.

Type	Items	Values
Input variables	C_1_ Salary	1 = Very dissatisfied 2 = Dissatisfied 3 = General 4 = Satisfied 5 = Very satisfied
	C_2_ Vacation	
	C_3_ Benefits	
	C_4_ Hours	
	C_5_ Flex‐schedule hours	
	C_6_ Straight days	
	C_7_ Weekends‐month	
	C_8_ Flex‐weekends off	
	C_9_ Compensation‐weekends	
	C_10_ Maternity leave	
	C_11_ Child care	
	C_12_ Supervisor	
	C_13_ Nursing peers	
	C_14_ Physicians	
	C_15_ Care method	
	C_16_ Social contact–work	
	C_17_ Social contact–after work	
	C_18_ Interact‐disciplines	
	C_19_ Interact‐faculty	
	C_20_ Belong‐department	
	C_21_ Control‐work setting	
	C_22_ Career advancement	
	C_23_ Recognition‐superiors	
	C_24_ Recognition‐peers	
	C_25_ Encouragement/feedback	
	C_26_ Participate research	
	C_27_ Write and publish	
	C_28_ Responsibility	
	C_29_ Control‐work conditions	
	C_30_ Decision‐making	

Output variable	Overall satisfaction (*D*)	1 = Very dissatisfied 2 = Dissatisfied 3 = General 4 = Satisfied 5 = Very satisfied

**TABLE 4:** Results of the IPA method for job satisfaction.

Items	Importance	Satisfaction	Classification
C_1_ Salary	0.010	3.516	III
C_2_ Vacation	0.005	3.391	III
C_3_ Benefits	0.005	3.647	III
C_4_ Hours	0.013	3.380	III
C_5_ Flex‐schedule hours	0.005	3.777	III
C_6_ Straight days	0.003	3.840	II
C_7_ Weekends‐month	0.009	3.579	III
C_8_ Flex‐weekends off	0.003	3.625	III
C_9_ Compensation‐weekends	0.005	3.348	III
C_10_ Maternity leave	0.015	3.837	I
C_11_ Child care	0.004	3.677	III
C_12_ Supervisor	0.007	4.082	II
C_13_ Nursing peers	0.002	4.277	II
C_14_ Physicians	0.001	4.071	II
C_15_ Care method	0.013	4.003	II
C_16_ Social contact–work	0.008	3.886	II
C_17_ Social contact–after work	0.013	4.027	II
C_18_ Interact‐disciplines	0.014	3.861	II
C_19_ Interact‐faculty	0.006	3.720	III
C_20_ Belong‐department	0.014	3.783	III
C_21_ Control‐work setting	0.018	3.916	I
C_22_ Career advancement	0.023	3.823	I
C_23_ Recognition‐superiors	0.009	3.970	II
C_24_ Recognition‐peers	0.028	4.033	I
C_25_ Encouragement/feedback	0.016	3.913	I
C_26_ Participate research	0.012	3.682	III
C_27_ Write and publish	0.005	3.609	III
C_28_ Responsibility	0.028	3.853	I
C_29_ Control‐work conditions	0.067	3.889	I
C_30_ Decision‐making	0.053	3.761	IV

*Note:* The importance and satisfaction of the center point are 0.014 and 3.792, respectively.

Figure 2 shows the importance of all input variables in descending order, with the Top‐5 being “Control‐work conditions (*C*_29_),” “Decision‐making (*C*_30_),” “Responsibility (*C*_28_),” “Recognition‐peers (*C*_24_),” and “Career advancement (*C*_22_)” and the last 5 being “Child care (*C*_11_),” “Straight days (*C*_6_),” “Flex‐weekends off (*C*_8_),” “Nursing peers (*C*_13_),” and “Physicians (*C*_14_).” Among the top five most important variables, “Control‐work conditions (*C*_29_)” and “Decision‐making (*C*_30_)” exhibited significantly higher importance compared to the others. These two variables are identified as the critical factors for improving overall nurses’ satisfaction in this case hospital.

### 4.3. Quadrant Categories of all Variables Using the IPA Method

**FIGURE 3:**
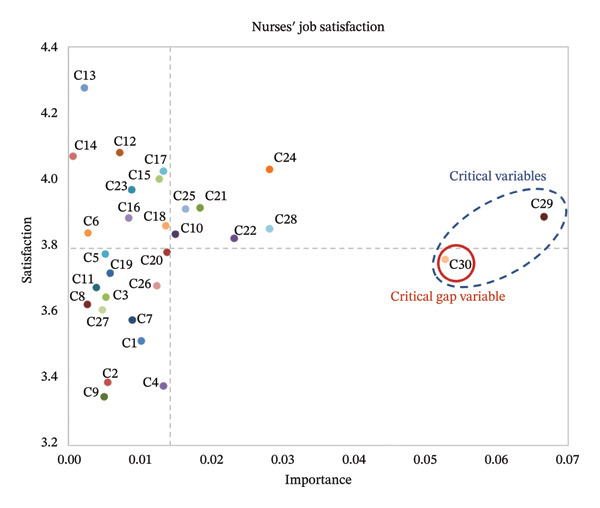
Analysis chart of the IPA method in the case hospital.

All input variables of the MMSS‐31 were divided into four quadrants based on their importance and satisfaction levels. The center points for importance and satisfaction were 0.014 and 3.792, respectively. The detailed data are shown in Table 4 and Figure 3, which are briefly described as follows:

The variables in Quadrant I are performing well at present and only require maintenance because they are of high importance and affect nurses’ overall satisfaction. Variables in this quadrant included “Maternity leave (*C*_10_),” “Control‐work setting (*C*_21_),” “Career advancement (*C*_22_),” “Recognition‐peers (*C*_24_),” “Encouragement/feedback (*C*_25_),” “Responsibility (*C*_28_),” and “Control‐work conditions (*C*_29_).”

The variables in Quadrant II are performing well at present and can therefore be considered without investing relevant resources for the time being, as they do not affect overall nurse satisfaction. Variables in this quadrant included “Straight days (*C*_6_),” “Supervisor (*C*_12_),” “Nursing peers (*C*_13_),” “Physicians (*C*_14_),” “Care method (*C*_15_),” “Social contact–work (*C*_16_),” “Social contact–after work (*C*_17_),” “Interact‐disciplines (*C*_18_),” and “Recognition‐superiors (*C*_23_).”

Variables in Quadrant III performed poorly at present; however, they can be ignored because they do not affect the overall satisfaction of nurses. Variables in this quadrant included “Salary (*C*_1_),” “Vacation (*C*_2_),” “Benefits (*C*_3_),” “Hours (*C*_4_),” “Flex‐schedule hours (*C*_5_),” “Weekends‐month (*C*_7_),” “Flex‐weekends off (*C*_8_),” “Compensation‐weekends (*C*_9_),” “Child care (*C*_11_),” “Interact‐faculty (*C*_19_),” “Belong‐department (*C*_20_),” “Participate research (*C*_26_),” and “Write and publish (*C*_27_).”

Variables in Quadrant IV performed poorly at present and therefore need to be prioritized because of their strong influence on the overall satisfaction of nurses. There is only one variable in this quadrant, i.e., “Decision‐making (*C*_30_).” At the same time, this variable is a priority for improvement. Based on the results of this case hospital, “Decision‐making (*C*_30_)” is considered a critical gap variable in this study.

## 5. Discussion

### 5.1. Critical Variables for Overall Satisfaction

Based on the importance results, it is clear that “Control‐work conditions (*C*_29_)” and “Decision‐making (*C*_30_)” are significantly more important than the other items, making them critical variables.

“Control‐work conditions (*C*_29_)” was ranked as a highly significant factor. It highlights the importance of workplace predictability, resource availability, and safety protocols. During infectious disease outbreaks, hospitals must maintain routine services. At the same time, they must strengthen infection control. Frontline nurses contributed significantly in both physical and emotional dimensions during COVID‐19. However, ongoing uncertainties have disrupted nurses’ control over their work environment [7]. New viral strains, shifting policies, and unpredictable shifts increase psychological pressure. Lack of clear schedules or inconsistent supply of PPE adds to stress. Nurses may feel powerless when they face unclear or unstable working conditions. The RF model shows that workplace stability is a critical predictor of job satisfaction. This includes consistent task assignment, clarity of responsibilities, and timely resource delivery. These elements often fluctuate during transitional pandemic stages. When poorly managed, they harm confidence, increase stress, and reduce satisfaction. Hospitals should aim to build predictable, well‐resourced environments to support frontline staff.

Similarly, “Decision‐making (*C*_30_)” was also ranked as highly significant. Nurses often face challenges in voicing their opinions or advocating for new ideas within their workplaces [45]. Hospital policies regarding prevention and control are heavily influenced by government directives and are frequently updated. Nurses have actively cooperated with government agencies and hospitals in the fight against the pandemic. However, as the COVID‐19 virus continues to mutate, nurses frequently encounter new and unfamiliar challenges while trying to maintain effective pandemic prevention and control measures. Therefore, having the opportunity to participate in decision‐making processes empowers nurses, enabling them to have a greater sense of control over their work environment and treatment strategies. Studies have also found that nurses’ involvement in decision‐making significantly affects job satisfaction [46, 47]. The pandemic has highlighted the necessity for shared decision‐making in both medical treatment and nursing care. Empowering nurses to contribute to infection control protocols or shift planning could not only improve satisfaction but also enhance operational efficiency. For instance, involving senior nurses in pandemic duty rotations reduces miscommunication and fosters ownership of outcomes. Beyond individual involvement, hospital administrators are encouraged to institutionalize participatory mechanisms during public health crises. This includes regularly organizing nurse roundtable meetings, establishing online suggestion portals, and inviting nurses from different hierarchical levels to participate in policy and workflow discussions. These inclusive practices not only improve transparency and mutual understanding but also give frontline staff a sense of agency, which is critical for boosting morale and job satisfaction during periods of uncertainty.

These findings are largely consistent with the results of previous studies, which have shown that nurses’ autonomy in decision‐making and their control over the work environment are directly related to occupational satisfaction and happiness [39]. Through the importance of RF, the input variables can be obtained from the data. This process revealed the underlying relationships between the output variables, allowing the identification and ranking of the potential importance of these input variables.

### 5.2. Critical Gap Variables of Nurse Satisfaction in This Case Hospital

The IPA analysis identified “Decision‐making (*C*_30_)” as a critical gap variable and the top‐priority area for improvement. This indicates that while decision‐making plays a crucial role in overall job satisfaction, nurses at the case hospital reported notably low satisfaction in this domain. In this study, decision‐making primarily refers to nurses’ participation in clinical and operational decisions, including patient care planning, workload distribution, and input on hospital‐level policies that directly impact nursing practice. In the context of this hospital, where epidemic prevention protocols and scheduling decisions are often made at the administrative level, enhancing bottom‐up engagement is essential. Hospital leaders and nursing managers should proactively collect feedback from frontline nurses and summarize relevant clinical insights during ongoing infectious disease control. Specific recommendations include the following: (1) establishing nurse roundtable meetings for regular input, (2) creating online suggestion portals, and (3) inviting nurses from various hierarchical levels to participate in decision‐related discussions. Furthermore, increasing opportunities for nurses to participate in real‐time work decisions can strengthen their psychological ownership and professional recognition. This inclusive approach is likely to foster a stronger sense of agency, thereby enhancing job satisfaction and organizational commitment among nursing staff.

### 5.3. Research Limitations

This study has certain limitations: (1) This study, in a single general hospital in Zhejiang Province with 368 nurses, offers valuable insights but may lack generalizability to other healthcare settings. Variations in administrative policies, staffing, and resource allocation could influence nurse satisfaction differently across public vs. private hospitals and urban vs. rural healthcare institutions. (2) The cross‐sectional design captures nurse satisfaction at a single time point, limiting its ability to analyze causal relationships or long‐term trends. (3) For all variables, the RF approach provides robust results for importance estimation. However, it is important to note that the importance of results may vary when applying different machine learning methods. The importance results and rankings presented here are based on the output of the RF approach.

### 5.4. Future Works

Based on the limitations of this study, several research directions can be considered for future studies: (1) Expanding the study population to include multiple hospitals across various regions would enhance the applicability of the findings. Comparative analyses between public vs. private hospitals, as well as cross‐cultural studies, could help identify both universal and region‐specific factors that influence nurse satisfaction. These insights would be valuable for developing tailored interventions that address the needs of diverse healthcare settings. (2) Future studies could consider adopting longitudinal approaches to track changes in nurse satisfaction over time, particularly in response to policy shifts or crisis situations. Longitudinal studies are crucial for monitoring trends in nurse satisfaction, especially in response to policy reforms, hospital management interventions, and healthcare crises such as pandemics. This would provide a more comprehensive understanding of the evolving factors that contribute to nurse satisfaction. (3) To improve predictive robustness and real‐world applicability, future research should explore more advanced machine learning techniques, such as deep learning and ensemble methods. These techniques could refine variable importance estimation and enhance pattern recognition in the determinants of nurse satisfaction, offering deeper insights into the factors that most significantly influence nurse well‐being in healthcare environments.

## 6. Conclusions

Based on the case hospital nurses’ experience, this study adopted the RF‐IPA hybrid model to explore the critical variables and critical gap variables in nurses’ job satisfaction during the dynamic adjustment phase of COVID‐19 prevention. Our findings indicate that decision‐makers and nursing departments at the case hospital should form a committee to integrate clinical nursing experiences relevant to the outbreak and to increase opportunities for clinical nurses to participate in decision‐making. This will enable them to adopt sensible strategies to improve job satisfaction in the face of unpredictable and large outbreaks in the future.

**Nomenclature**
RF: Random forest
IPA: Important‐performance analysis
MMSS‐31: McCloskey/Mueller Satisfaction Scale–31 items

## Author Contributions

Weifang Xu, Lili Feng, and Yen‐Ching Chuang conducted the study and drafted the manuscript. Siqi Liu and Mao Ye conducted a questionnaire survey and data collection. Yen‐Ching Chuang analyzed the data and used the R language/software. Fuqin Tang and Mao Ye conceived the study and participated in its design and coordination. Weifang Xu, Chao Liu, and Yen‐Ching Chuang participated in the revision process of the manuscript.

## Funding

This work was supported by the grants from the Zhejiang Medical and Health Science and Technology Program (Grant no. 2023KY1337); the Science and Technology Planning Project of Taizhou City, Zhejiang Province (Grant IDs: 21ywb39 and 22ywb30); the Education Planning Project of Taizhou City, Zhejiang Province (Grant ID: GG22016); and the Nursing Discipline Development Special Fund Project of Taizhou University, Zhejiang Province (Grant ID: 202201).

## Disclosure

All authors read and approved the final manuscript.

## Ethics Statement

This study was reviewed and approved by the Ethics Committee of Taizhou Central Hospital (Taizhou University Hospital) under approval number: 2022L‐09‐28. All procedures involving human participants were conducted in accordance with the ethical standards of the institutional research committee and the principles outlined in the Declaration of Helsinki. Written informed consent was waived by the ethics committee due to the survey’s noninterventional, anonymous nature. Prior to participation, all respondents were fully informed about the purpose and content of the study, and voluntary completion of the questionnaire was considered to constitute oral informed consent.

## Consent

Please see the Ethics Statement.

## Conflicts of Interest

The authors declare no conflicts of interest.

## Data Availability

The datasets generated and/or analyzed during the current study are available from the corresponding author on request.
